# Imaging of metastatic epidural spinal cord compression

**DOI:** 10.3389/fradi.2022.962797

**Published:** 2022-08-12

**Authors:** James Bai, Kinzya Grant, Amira Hussien, Daniel Kawakyu-O'Connor

**Affiliations:** Department of Imaging Sciences, University of Rochester, Rochester, NY, United States

**Keywords:** metastatic epidural spinal cord compression (MESCC), spine, metastatic disease, spinal cord compression, spine imaging

## Abstract

Metastatic epidural spinal cord compression develops in 5–10% of patients with cancer and is becoming more common as advancement in cancer treatment prolongs survival in patients with cancer (1–3). It represents an oncological emergency as metastatic epidural compression in adjacent neural structures, including the spinal cord and cauda equina, and exiting nerve roots may result in irreversible neurological deficits, pain, and spinal instability. Although management of metastatic epidural spinal cord compression remains palliative, early diagnosis and intervention may improve outcomes by preserving neurological function, stabilizing the vertebral column, and achieving localized tumor and pain control. Imaging serves an essential role in early diagnosis of metastatic epidural spinal cord compression, evaluation of the degree of spinal cord compression and extent of tumor burden, and preoperative planning. This review focuses on imaging features and techniques for diagnosing metastatic epidural spinal cord compression, differential diagnosis, and management guidelines.

## Introduction

Metastatic disease of the spine is an increasingly common complication of malignancy as advances in cancer management have increased life expectancy following the initial diagnosis of cancer. Epidural spinal cord compression caused by lesions is a source of significant morbidity, including neuropathic pain and loss of neurologic function, resulting in significant reduction in patient quality of life. Early clinical recognition of epidural disease is critical in effective treatment, as the severity and duration of neurologic deficit prior to initiation of treatment are key prognostic factors for degree of recovery (4, 5). Both diagnosis and management rely heavily on the use of advanced imaging techniques and allow for differentiation from non-neoplastic etiologies of spine disease. Contrast-enhanced MRI (CE-MRI) presently represents the highest available care standard for detecting and characterizing epidural lesions, and for guiding management, which may include surgical decompression and stabilization, radiotherapy, and medical management. This review will provide an up-to-date overview of imaging techniques for identifying and characterizing causes of metastatic epidural cord compression and related differential diagnoses, and a brief review of management guidelines.

## Epidemiology

Approximately 5–10 % of patients with cancer develop metastatic epidural spinal cord compression (1, 2). Epidural metastases may originate from many types of cancers: breast, lung, and prostate cancer each account for 10–20% of cases; renal cell carcinoma, lymphoma, multiple myeloma each account for 5–10% of cases; and colorectal cancer, sarcoma, and cancer of unknown origin account for the remainder (6–8). In pediatric patients, epidural metastases are most commonly from Ewing's sarcoma and neuroblastoma, and less commonly from osteogenic sarcoma, rhabdomyosarcoma, Hodgkin's disease, germ-cell tumor, and Wilm's tumor (9, 10).

It is estimated that 85% of epidural metastases originate from local invasion from adjacent spinal osseous metastasis, more often arising from the vertebral column than the posterior neural arch (3). In addition, 10–15% of cases originate from paraspinal soft tissues and pass through neuroforamina, and are commonly associated with lymphoma (3). Isolated metastases to the epidural space are rare but usually occur by hematogenous dissemination from the venous plexus of Batson or radicular arterioles (3, 11).

Epidural spinal metastases are more common in the thoracic spine (68%) than the lumbar (16%) and cervical spine (15%), and the majority of tumors occupy the anterior portion of the spinal canal (3, 11, 12). The site of compression is generally related to bone mass, blood flow, and spinal canal caliber (7, 12).

Epidural metastatic spinal cord compression usually develops in the late stage of malignancy and generally occurs in patients with a preexisting diagnosis of cancer. However, it is the initial manifestation of malignancy in 20% of cases (13). In patients with lung cancer, up to 30% of epidural metastatic cord compression develops in patients without a preexisting history of cancer (13).

## Spinal canal compartmental anatomy

Understanding spinal canal compartmental anatomy is important for lesion localization. Metastases to the spine localize to the epidural, leptomeningeal, or intramedullary space.

The three meningeal layers, dura mater, arachnoid mater, and pia mater, encase the spinal cord, cauda equina, spinal nerve roots, and their vascular supply. The dura mater is the outermost meninx that attaches cranially to the periosteum of the foramen magnum and terminates caudally as the coccygeal ligament, the layer of dura mater which encases the filum terminale (14). The arachnoid mater is a thin transparent layer of meninx that attaches to the inner dura mater without intervening physiological subdural space. Cranially, it is continuous with the cranial subarachnoid space at the foramen magnum, and caudally it encloses the nerve bundles of the cauda equina. It terminates between the first and second sacral vertebrae (14). The pia mater is a thin transparent layer that adheres to the spinal cord, is contiguous with the cranial pia mater at the foramen magnum cranially, and terminates caudally as a thin ligament known as filum terminale.

**Figure 1:**
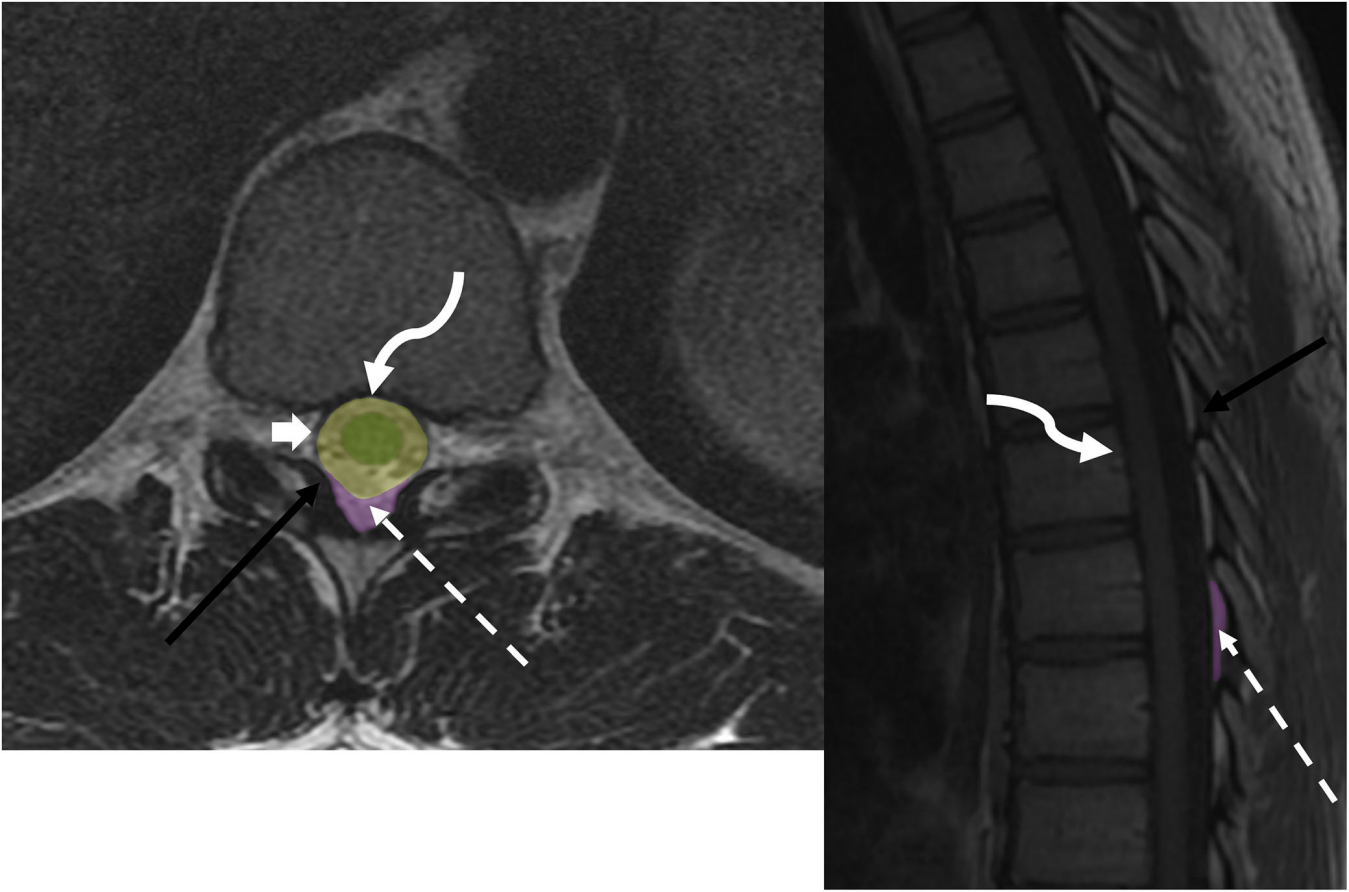
Anatomy of epidural space: axial T2w and sagittal T1w sequences demonstrate thecal sac (broad white arrow), ligamentum flavum (black arrow), epidural fat (dotted white arrow), and posterior longitudinal ligament (curved arrow). Spine compartmental anatomy includes the intramedullary space (green), intradural space (yellow), and dorsal epidural space (purple).

The epidural space is defined as the space between the dura mater of the spinal cord and the overlying ligamentous structures of the spinal canal, specifically the posterior longitudinal ligament anteriorly and the ligamentum flavum posteriorly (Figure 1). The epidural space contains epidural fat, venous plexus, lymphatics, small arterioles, and spinal nerves. The subdural space is a potential space between the dura mater and the arachnoid mater, which is not present under normal physiological conditions. Fragile collagens attach the two layers, which can be dissected without opening the subarachnoid space (14). The subarachnoid space is a CSF-filled space between the two innermost leptomeninges, the arachnoid and pia mater, and is bridged by a network of web-like connective trabeculae. The intramedullary space contains substances of the spinal cord encased by the pia mater.

**Figure 2:**
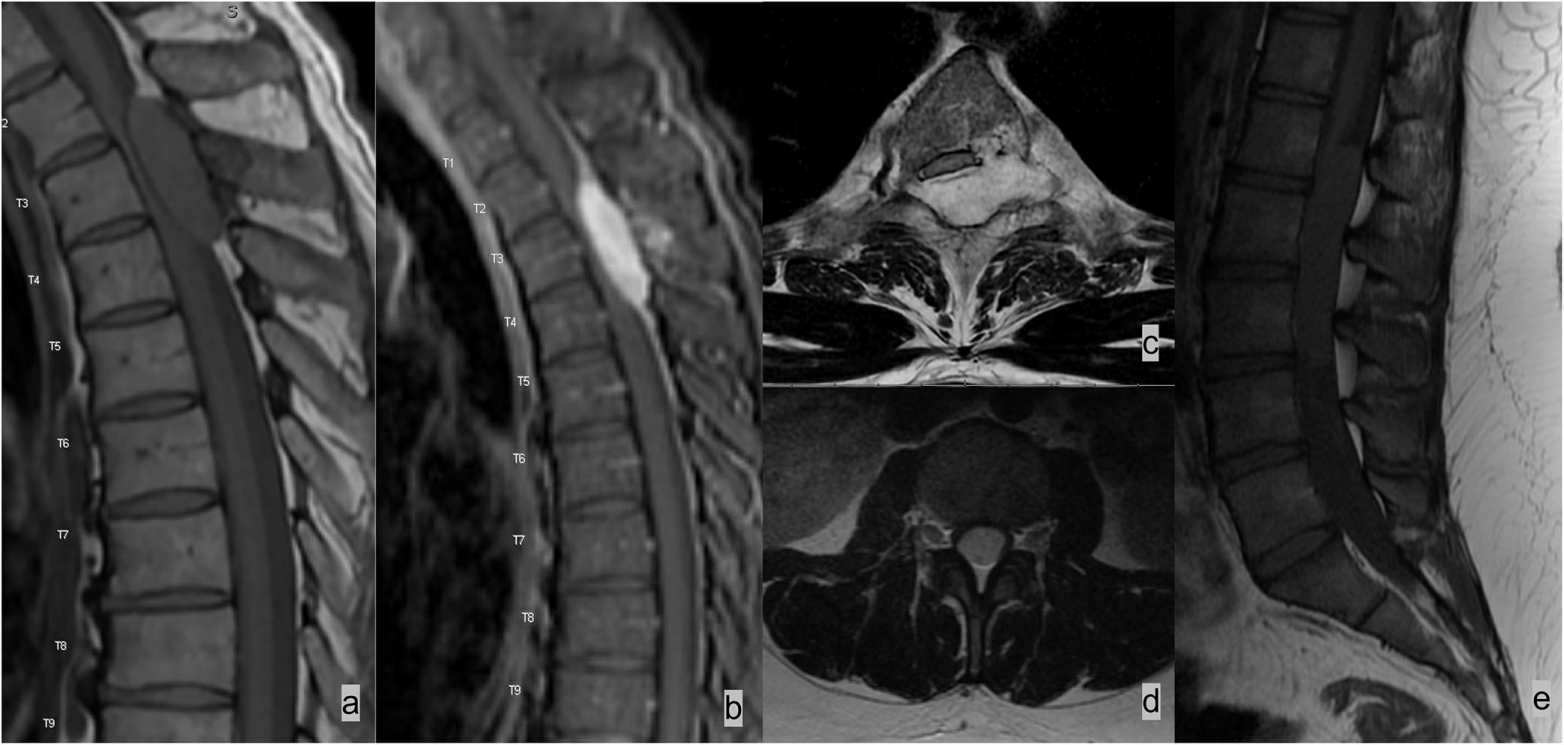
Two different patients with **(a–c)** epidural and **(d,e)** intradural spinal masses. **(a–c)** Patient 1 had spinal epidural hemangioma. MRI demonstrated a T2 hyperintense enhancing mass in the left dorsal and lateral epidural spaces compressing and displacing the spinal cord. Lesions in the epidural space results in effacement of the epidural fat and inward displacement of T1 and T2 hypointense thecal sac. **(c,d)** Patient 2 had myxopapillary ependymoma, an intradural mass, which expands the dural sac and compresses cauda equina nerve bundles. The epidural fat is preserved in an intradural lesion.

**Figure 3:**
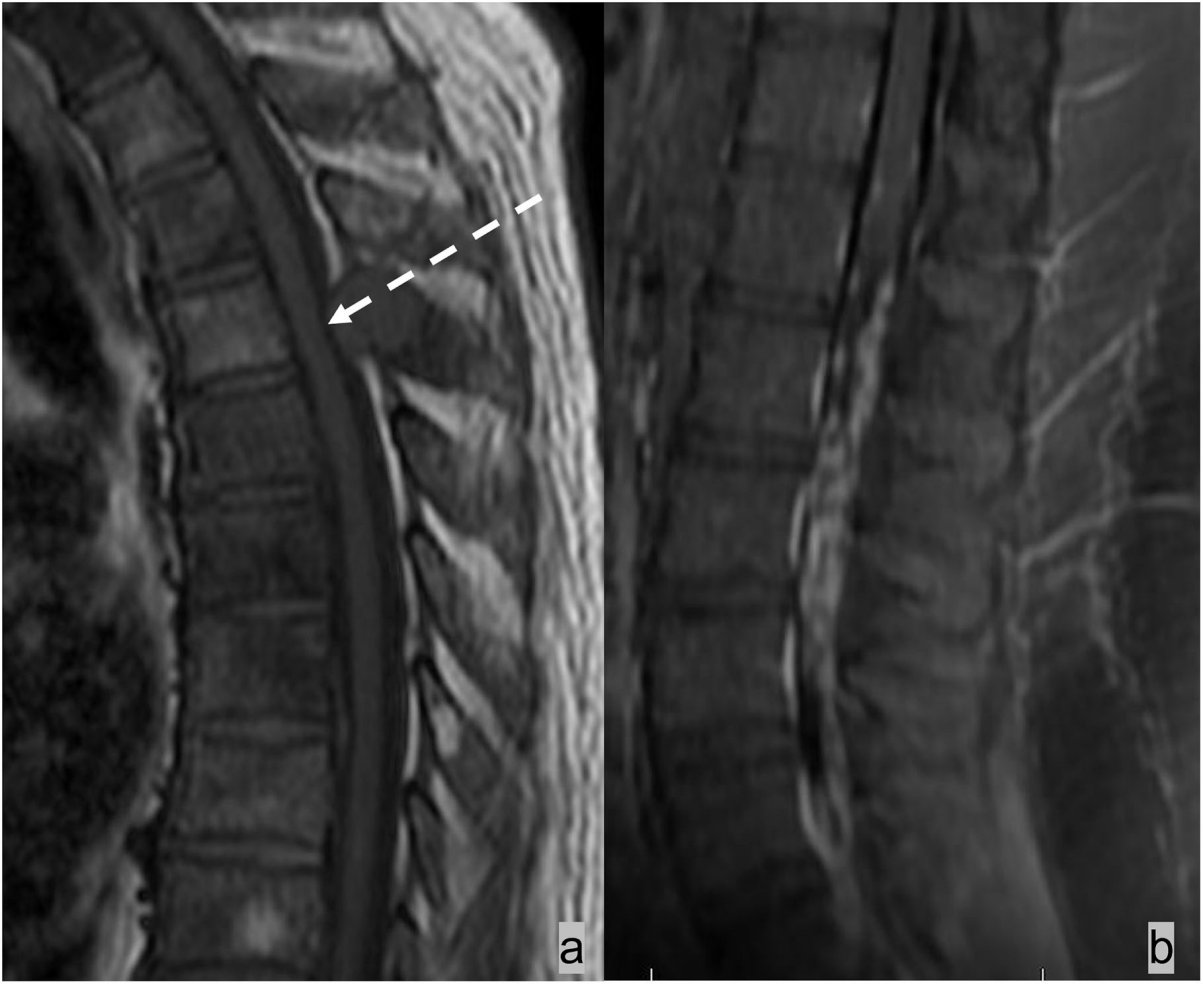
Two different patients with **(a)** epidural and **(b)** intradural leptomeningeal metastases. **(a)** Patient 1 had lung cancer with spinal metastases. Sagittal T1w sequence demonstrated multiple marrow-replacing bony metastases. The metastatic lesion at the T4 spinous process (white arrow) effaces the epidural fat and results in inward displacement of the thecal sac. **(b)** Patient 2 had neoplastic meningitis from metastatic lymphoma. MRI of the lumbar spine demonstrated extensive nodular leptomeningeal enhancement on the post-contrast T1w image.

On MR imaging, an epidural lesion effaces the epidural fat, causes inward displacement of T1 and T2 hypointense dura mater, and compresses the thecal sac (Figures 2, 3). An intradural lesion is in the thecal sac without inward displacement of the dura mater and preserves the epidural fat (Figures 2, 3). A CSF cleft can be seen between an intradural lesion and the spinal cord (15). An intramedullary lesion is centered within and expands the spinal cord.

## Pathophysiology

Spinal bony metastases are the primary source of metastatic epidural spinal cord compression. The vertebral column is the most common site of skeletal metastases, primarily disseminated through hematogenous spread (3, 6–8, 10–12, 16–20). Factors contributing to the high incidence of bony metastases in the vertebral column include vertebral vascular anatomy and the abundance of growth factors in the hematopoietic bone marrow.

The valveless Batson's epidural venous plexus has bidirectional flow, which increases the chance for tumor cell seeding as activities that elevate intrathoracic and intra-abdominopelvic pressure such as coughing and Valsalva maneuver can lead to venous reflux draining from visceral organs (3, 17, 21). In addition, due to the paucity of vertebral arterioles anastomosis, the distal arterioles functionally act as terminal arterioles, which can entrap cancer cells and facilitate hematogenous dissemination (17).

The hematopoietic bone marrow in the spine has abundant growth factors and high blood flow, which provide both a physical niche and nutritional support for cancer cells (12, 17, 22, 23). Bony invasion by tumor cells is associated with osteoclast and osteoblast recruitment by tumor cells, which release growth factors from the bone matrix and feedback to tumor growth and self-perpetuate into a vicious cycle (23, 24). Osteoclasts are cells that promote demineralization and bone resorption by degradation of the newly formed extracellular matrix (ECM) by secreting protease (24). Osteoblasts promote bone reconstruction by generating new ECM and deposition of calcium phosphate to the matrix (24). Osteolytic predominant bony invasions such as in breast cancer, lung cancer, and renal cell carcinoma, promote osteoclastic activities and are maintained by mitogenic factors of tumor cells such as transforming growth factor (TGF-β), insulin-like growth factor-1, parathyroid hormone-related peptide (PTHrP), fibroblast growth factors (FGFs), and platelet-derived growth factors (PDFGs) (24). Osteoblastic predominant osseous metastasis, mainly prostate cancer, has a tumor microenvironment that accumulates immature mineralized bone (osteoid), which leads to osteosclerosis (24). Osteolytic metastases are generally more aggressive and manifest early clinical presentation because of demineralization and bone resorption promoted by osteoclasts (25).

The process of epidural metastases that leads to spinal cord compression is a complex but predictable event. Cancer cells replace and expand the hematopoietic bone marrow and subsequently spread to the epidural space and spinal canal through basivertebral veins and other penetrating vessels. Cancer cells subsequently proliferate and form tissue masses in emergent sites, most commonly along the anterior spinal canal. More infiltrating tumor cells can migrate to the posterior neural arch and form tissue masses in the epidural space of the posterior spinal canal (12). The tissue mass effect on the spinal cord leads to mechanical obstruction of spinal blood flow, which results in venous congestion, breakdown of the spinal cord-blood barrier, and spinal cord edema, and eventually progresses to irreversible ischemia, hemorrhage, and finally necrosis (12). The histologic evidence from animal studies demonstrated that cortical destruction occurs in a later stage (17). Bony destruction eventually contributes to pathological fracture and results in mechanical instability and compression of the spinal cord or nerve bundles of the cauda equina.

## Clinical presentations

Back pain is the earliest and most common presentation in patients with metastatic epidural spinal cord compression (3, 7, 16), and 95% of adult patients (11) and 80% of pediatric patients (26) have initial back pain symptoms. Back pain from metastatic disease can manifest as localized pain from stretching the periosteum, radicular pain from compression on nerve roots, or mechanical pain from mechanical instability due to pathological fracture (7). Back pain, however, is a nonspecific symptom and commonly occurs in degenerative diseases. However, the pain of metastatic epidural compression can occur at any level, whereas degenerative disease most commonly involves the lower cervical and lower lumbar regions (3, 27).

Weakness is the second most common symptom and can involve both upper or lower motor neurons depending on the location (7). Sensory deficit rarely occurs before pain or motor deficit (7). Autonomic symptoms present in the late stage of the disease. Isolated bowel and bladder dysfunction are rare without other symptoms (10). Isolated ataxia without pain or motor deficits can develop in patients with metastatic epidural compression in the spinocerebellar tract (11).

## Imaging techniques and features

MRI is the imaging of choice for diagnosing metastatic epidural spinal cord compression. MRI has excellent soft tissue contrast resolution, which allows for accurate detection and assessment of metastatic marrow infiltration, epidural and transforaminal tumor extension, paraspinal soft tissue involvement, and degree of spinal cord compression. CT is superior in delineating the osseous cortex, which aids in evaluating cortical integrity. CT myelography is a high-resolution adjunct or alternative imaging modality for assessment of epidural spinal cord compression and transforaminal extension, and is the examination of choice for patients with contraindications for MRI such as incompatible implants, movement disorders, and claustrophobia. Nuclear bone scintigraphy is highly sensitive for detecting osteoblastic lesions and aiding early detection and evaluation of the extent of bony metastases. [18F]-fluoro-2-deoxy-d-glucose positron emission tomography (FDG-PET) CT or MRI combines functional and structural imaging data to provide early detection, identification of primary neoplasm of unknown origin, and evaluation of treatment response.

### MR imaging

When there is a clinical suspicion of metastatic epidural spinal cord compression, contrast-enhanced MRI is the imaging modality of choice. MRI has a superior contrast resolution that allows to distinguish pathological tissues from normal tissues. Contrast is needed for assessing epidural and paraspinal soft tissue extension and increases the sensitivity of detection of intraosseous metastases. Multilevel metastatic epidural disease occurs in 20–35% of patients with epidural spinal metastases (7, 18, 28); imaging of the entire spine is thus necessary to identify additional spinal metastases.

Conventional MRI sequences, including T1-weighted (T1w), T2-weighted (T2w), and Short Tau Inversion Recovery (STIR) sequences, are highly accurate for detecting spinal osseous metastases (98.5% sensitivity and 98.9% sensitive) (19, 29). STIR sequence is a fat suppression technique using an initial 180° radiofrequency (RF) pulse inverting spins in the longitudinal direction and achieving fat suppression by applying an initial 90° excitation RF pulse when the longitudinal spin of fat is zero (30). STIR uniformly suppresses fatty marrow signals and thus enhances the conspicuity of marrow replacing intraosseous neoplastic lesions. It is a fat suppression technique relatively independent of magnetic field inhomogeneity and can be performed on virtually any MRI machine. It also has an advantage over other fat saturation techniques in the presence of surgical hardware and metallic foreign body. STIR sequences, when interpreted with T1w sequences, are highly accurate in identifying intraosseous metastases (30). Another fat suppression technique, the DIXON method, has gained interest in spinal imaging in recent years. It achieves fat suppression using the chemical shift principle by acquiring in-phase and out-of-phase images, which can be post-processed into water-only and fat-only sequences. The advantages of the DIXON technique include homogeneous and reliable fat suppression. In addition, four sequences with and without fat suppression with one acquisition allow for better characterization of fat. It is more versatile and can be used with gradient-echo and spin-echo sequences and with different weightings, including T1, T2, and proton density (31, 32). The DIXON technique is, however, more susceptible to metal artifacts and has longer acquisition time than STIR using the same parameters (32).

Spinal intraosseous metastases are marrow-replacing lesions with low signal intensity on the T1w sequence and hyperintensity on the STIR sequence or other fat-suppressed T2w sequence. They can have variable signal intensities on the non-fat-suppressed T2w sequence. Sclerotic lesions such as prostate metastases have hypointense signals on the T1w and T2w sequences. Metastatic lesions containing intrinsic T1 hyperintense materials, such as melanin, hemorrhage, or protein aceous material, have intrinsic hyperintense signals similar to the fat signals on the nonfat-suppressed T1w sequence. These lesions are more conspicuous on fat-suppressed sequences.

Post-traumatic marrow inflammation and edema, degenerative changes, reactive Schmori's nodes, and normal marrow variants, such as red marrow islands and focal nodular marrow hyperplasia, can mimic intraosseous metastases by generating abnormal marrow edema signals on the fat-suppressing sequence and hypointensity relative to bone marrow on the T1w sequence. In contrast to aggressive neoplastic tissues, these benign lesions do not replace the marrow, still preserve some fat content, and therefore generate more T1 signals in comparison with fat replacing neoplasm on the T1w sequence (31). It is helpful to compare with the internal standard, including muscle and normal disc material, on T1w sequences to distinguish benign lesions from fat-infiltrating pathology (31, 33–35).

**Figure 4:**
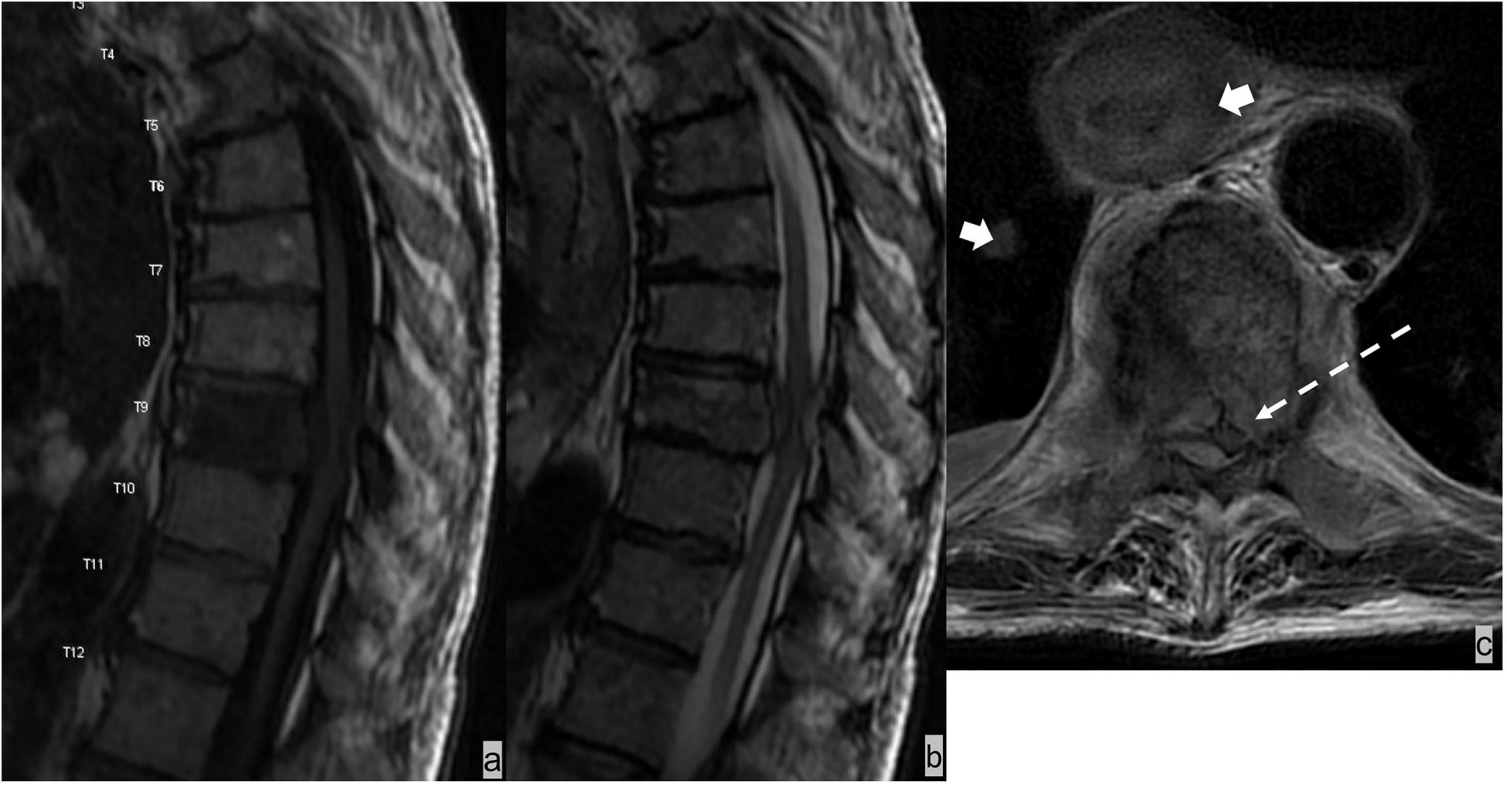
The patient had esophageal cancer with spinal metastases. Sagittal T1w and T2w sequences of the MRI lumbar spine **(a,b)** demonstrated marrow-replacing bony metastases with ventral and dorsal epidural extension (dotted white arrow), complete effacement of the thecal sac, and spinal cord compression at T9. This is a grade 3 epidural cord compression on the ESCC scale **(c)**. MRI also showed mural thickening of the esophagus and lung metastases (broad white arrow).

**Figure 5:**
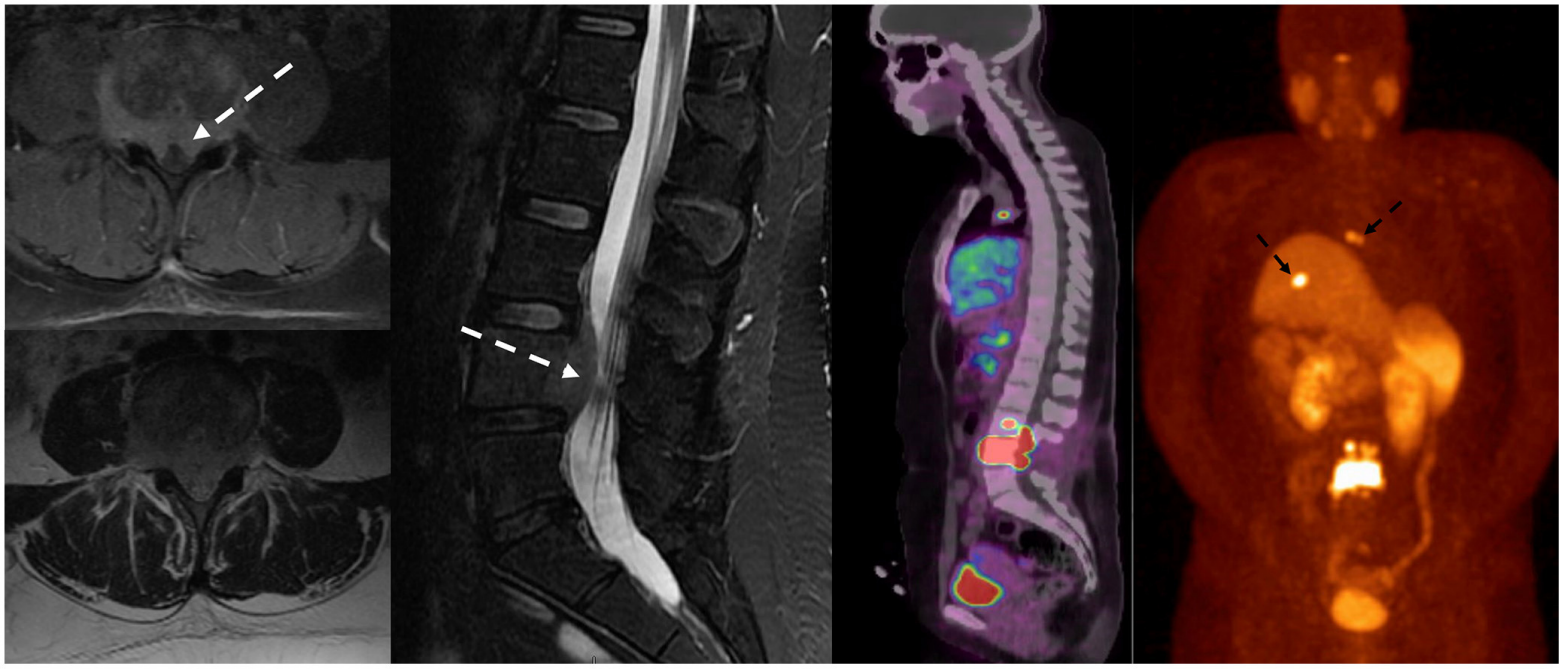
Patient with a history of lung carcinoid tumor status post-surgical resection presented with back pain. MRI of the lumbar spine demonstrated lumbar spinal metastasis with ventral epidural extension partially effacing the thecal sac at L4 (dotted white arrow). Ga-68 DOTATATE PET/CT showed avid DOTATATE uptake of the lumbar spinal metastasis and additional metastatic lesions in the right peritracheal lymph node and the liver (dotted black arrow).

**Figure 6:**
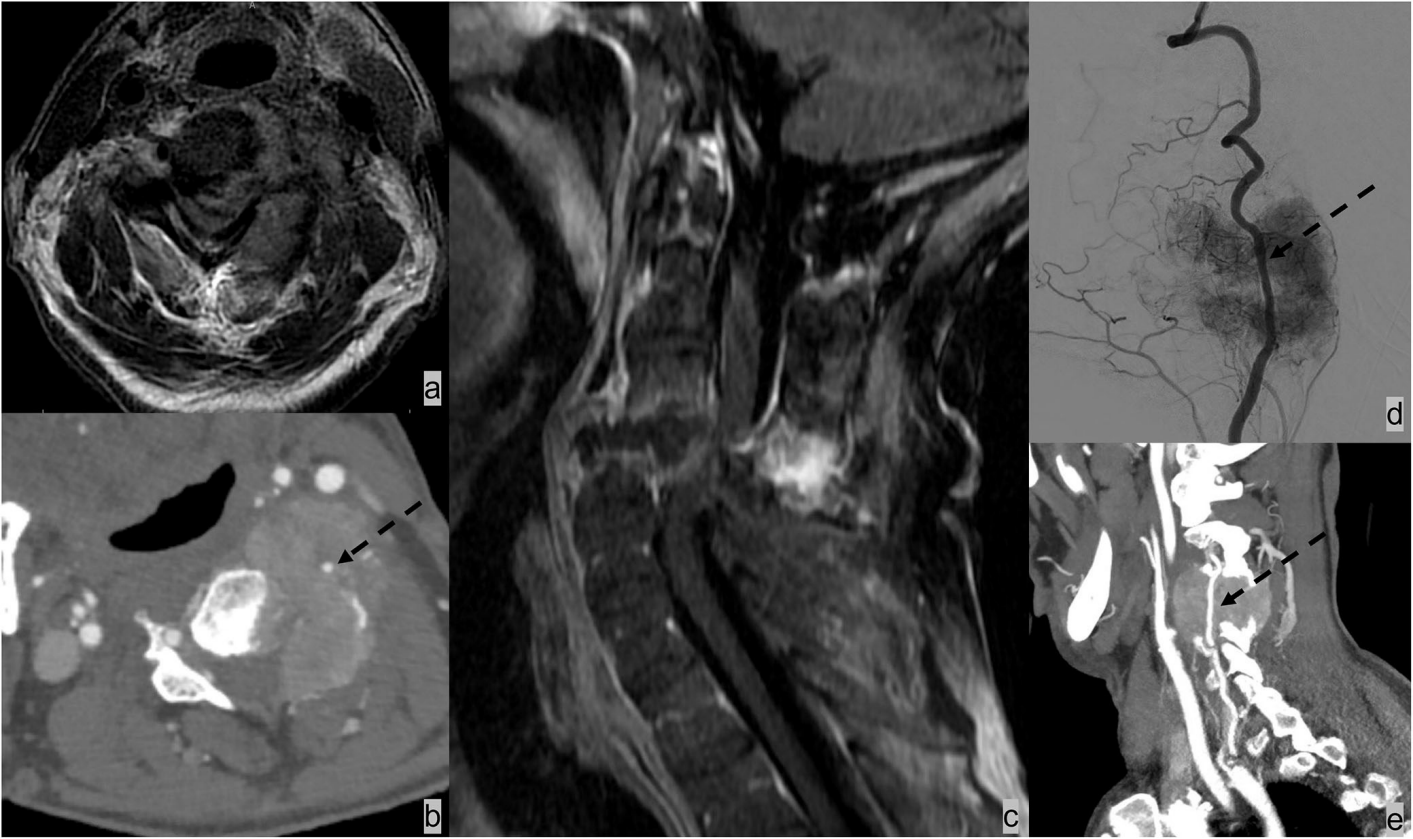
Patient had renal cell carcinoma with spinal metastases. **(a–c)** MRI and CT angiogram neck demonstrated osseous metastasis with soft tissue extending the ventral lateral epidural space and paraspinal space and resulting in complete effacement of the thecal sac and spinal cord compression. **(b)** It is further complicated by pathological fracture of C6 and posterior subluxation that resulted in mechanical instability. The metastatic lesion encases the left vertebral artery [dotted black arrow; **(b,d,e)**]. The patient underwent selective tumor embolization before surgical debulking and stabilization. Digital subtraction angiography demonstrates extensive tumor brush **(d)**.

**Figure 7:**
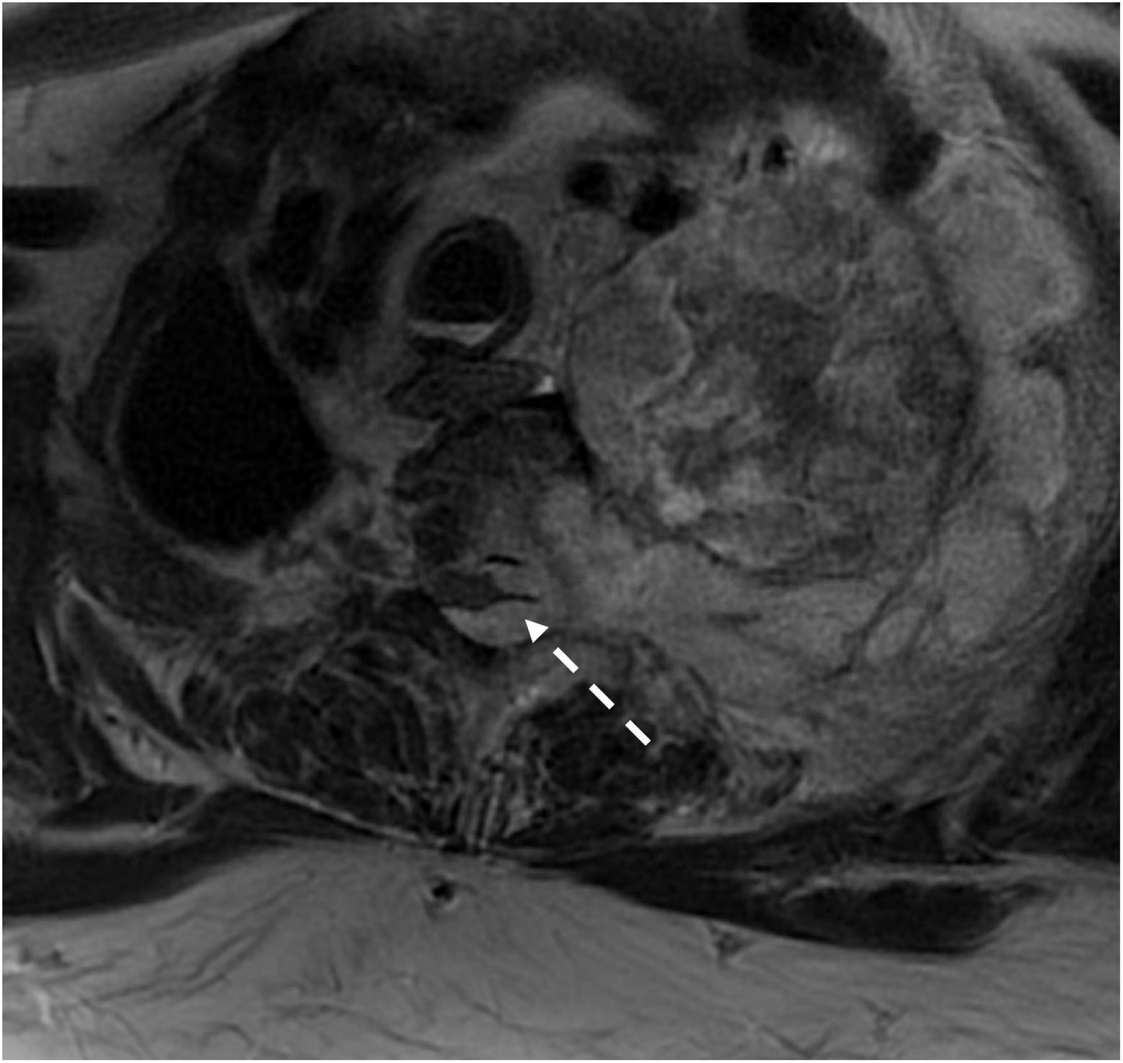
Patient with type 1 neurofibroma presented with a large paraspinal soft tissue mass with intrathoracic and epidural extension and cord compression. Surgical pathology demonstrated malignant transformation of the nerve sheath tumor.

**Figure 8:**
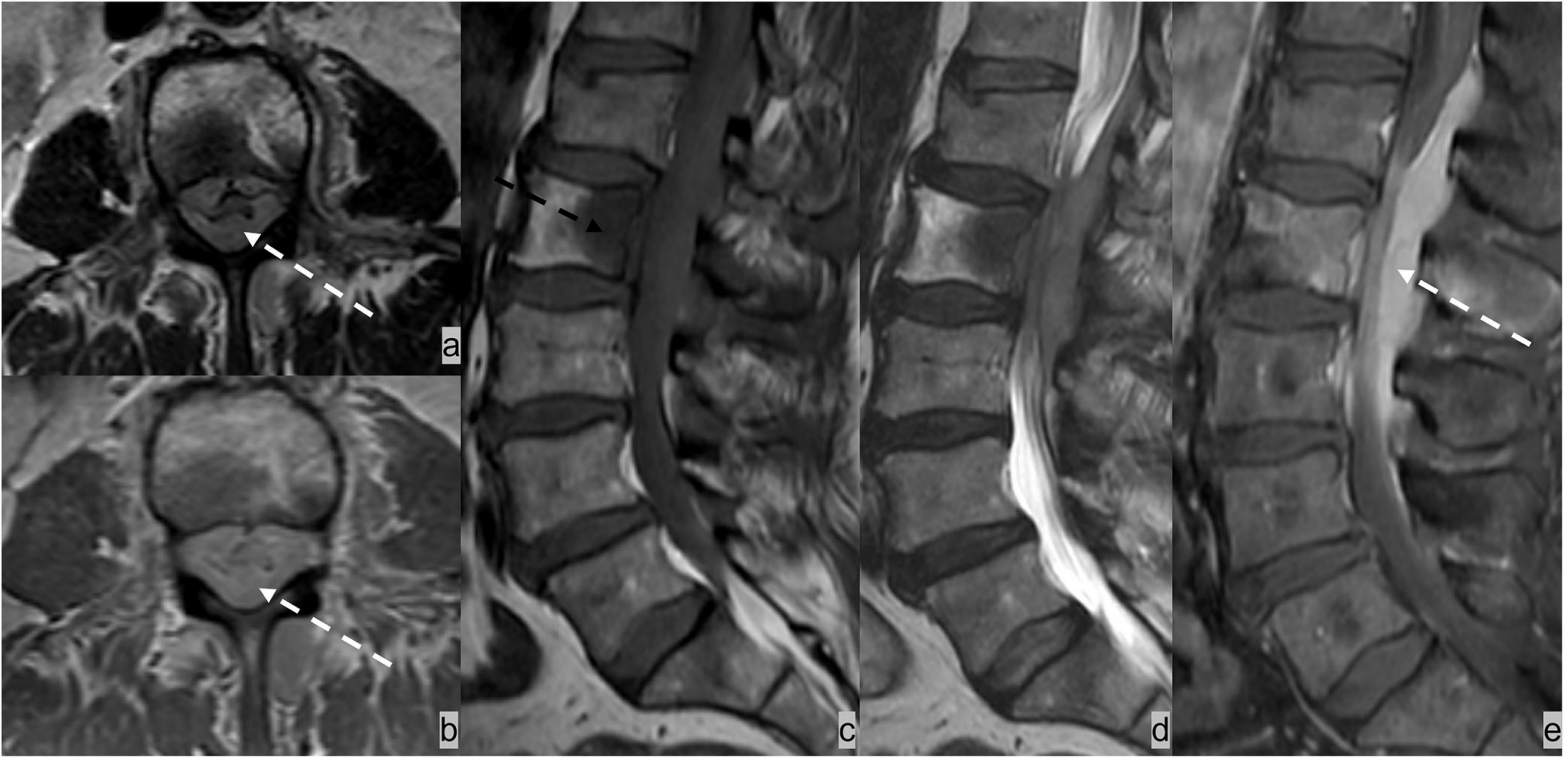
Forty-five-year-old patient presented with cauda equina syndrome including burning pain in the bilateral lower extremities, weakness, and urinary retention. **(a)** AxialT2w, **(b)** axial post-contrast T1w, **(c)** sagittal T1w and **(d)** T2w, and **(e)** sagittal post-contrast T1w MRI sequences demonstrated an enhancing T1 hypointense and T2 iso-hypointense osseous lesion involving L2 vertebrae (dotted black arrow) and an enhancing epidural soft tissue completely encasing the thecal sac and compressing cauda equina nerve bundles (dotted white arrow). Biopsy of the L2 lesion showed germinal center-type diffuse large B cell lymphoma.

Epidural tumor extension generally demonstrates T1 hypointensity, variable T2 signals, and contrast enhancement (Figures 4–8). Post-contrast T1w sequences are the best sequence for localizing and assessing epidural tumor extension. Epidural metastases are generally contrast-enhancing, although regions of tumor necrosis can demonstrate areas of non-enhancement. In the presence of spinal canal stenosis from other causes such as degenerative spondylosis, congested venous plexus can show contrast enhancement and mimics tumor extension in the ventral epidural space (36).

T2w is the most sensitive sequence for assessing spinal cord signal abnormality and the extent of spinal cord compression and displacement (Figures 4–7) (37). With its high T2 signal, CSF provides excellent background contrast for the spinal cord and the extrinsic lesion that compresses the spinal cord. The sagittal T2w sequence gives a panoramic overview of the spinal segment for assessing the spinal cord's morphology, course, size, and signal. With its small field of view, the axial T2w sequence offers a more detailed assessment of the presence and extent of spinal cord compression, displacement, and intrinsic cord signal abnormality. Compression of the ventral spinal canal can result in epidural venous plexus congestion, leading to spinal cord blood barrier breakdown and parenchymal edema of the spinal cord. Spinal cord edema shows ill-defined, often expansile T2 signal abnormality on imaging. Subsequently, spinal cord compression can impair arterial supply, resulting in cord ischemia and infarction, which causes irreversible neurological damage. Spinal cord ischemia and edema demonstrate T2 signal hyperintensity and restricted diffusion. In severe compression myelopathy, typical T2 signal hyperintensity may not be evident at the maximal level of compression but rather present in the adjacent segment (36). In the chronic phase of cord compression, T2 signal abnormality becomes more well-defined, and volume loss becomes evident because of myelomalacia and gliosis.

### Computed tomography (CT)

CT has excellent delineation of bony cortex and trabeculation, and therefore is helpful for evaluating cortical integrity, periosteal reaction, and destructive changes by aggressive lesion vs. bony remodeling by a more chronic process (19). CT has low sensitivity for detecting intraosseous metastases without bony destruction (66.2%) (29). The overall diagnostic accuracy of MRI in detecting spinal osseous metastases is higher than that of 16/64 row MDCT (98.7 vs. 88.8%) (29).

Recent studies on dual-energy CT (DECT) imaging have shown promising early results for detecting bone marrow infiltration. Kosmala et al. reported 91.3% sensitivity and 90.9% specificity of DECT in detecting marrow infiltration in patients with multiple myeloma using the virtual non-calcium technique (38).

CT has a limited role in detecting early-stage epidural metastases and paraspinal soft tissue extension. Late-stage epidural metastases on CT often show ill-defined soft-tissue attenuating epidural and transforaminal mass with adjacent osseous destruction (Figure 6b). These findings, however, can be obscured due to beam hardening arising from the dense cortical bone of the spine. Despite MRI has superior overall performance for evaluation of spinal metastatic disease, CT remains an important and complementary alternative to MRI due to fast-scanning time, low cost, high availability, and excellent characterization of cortical bone.

CT myelography may be performed in conjunction with MRI according to preferences of referring surgeons, or as an alternative imaging modality in scenarios in which MRI is contraindicated. Relative contraindications include patients with movement disorders or those who are otherwise unable to remain sufficiently stationary for the duration of an MRI scan. Absolute contraindications include the presence of medical devices that are incompatible with MR imaging including ventricular assist devices, cardiac pacemakers, defibrillators, neurostimulator devices, drug infusion pumps, and certain ferromagnetic or recently deployed vascular clips and stents. Similarly, retained foreign bodies, such as projectile fragments, particularly metallic fragments in the orbit or spinal canal are also considered absolute contraindications. Much like MRI, CT myelography may be performed to evaluate spinal canal contents, including configuration of the thecal sac, canal stenosis, and degree of spinal cord compression (39). In addition to providing important diagnostic information when MRI findings are inconclusive, CSF samples can be obtained for laboratory analyses when performing CT myelogram. CT myelogram is also routinely conducted in treatment planning for stereotactic body radiotherapy (SBRT) (19).

### Nuclear imaging

Bone scintigraphy is a widely used screening method for initial assessment of bony metastases. Whole-body imaging, high availability, relatively low radiation, and low cost are all advantages as a routine screening tool. Tc-99m-methylene diphosphonate (MDP) is the most used tracer, which accumulates in bony metastases during osteoblastic activities by chemosorption. Imaging detection depends on the degree of osteoblastic activity and degree of blood flow (40). Most osseous metastases have mixed osteoblastic and osteolytic activities, both of which demonstrate increased tracer uptake on bone scan. However, bone lesions with complete absence of osteoblastic response or poor blood flow can result in false negatives. Multiple myeloma, leukemia, and anaplastic carcinoma have the highest false-negative rates (40). Widespread osseous metastases can have a diffuse accumulation of tracer uptake throughout skeletal structures (super scan), most commonly in prostate cancer metastases, and could be misinterpreted as a negative scan (40). Tracer uptake in a bone scan is not highly specific for osseous metastases as tracers can also accumulate in healing fracture, infection, inflammation, and hypertrophic degenerative changes. Solitary lesions detected by bone scan are nonspecific, and only 50% represents osseous metastases (40, 41). Single-photon emission computed tomography (SPECT) has improved sensitivity and specificity compared to planar images (42, 43), as aggressive bony changes can be better localized and characterized on the SPECT acquisition.

[18F]-fluoro-2-deoxy-d-glucose positron emission tomography (FDG-PET) CT or MRI combines functional and structural imaging data, providing a powerful oncological imaging tool. FDG is a highly sensitive but nonspecific PET tracer detecting glucose hypermetabolism in tumor cells and is therefore a primary oncological imaging tool for identifying neoplasm of unknown origin, cancer staging, and evaluation of treatment response. The diagnostic accuracy of 18-F-FDG-PET/CT is dependent on tumor biology. Sensitivity for lytic metastases is generally higher than for osteoblastic metastases because of higher FDG uptake by lytic metastases (44). A meta-analysis of lung cancer metastases in bone demonstrated overall better sensitivity and specificity of 18F-FDG-PET/CT (92 and 98%) compared to MRI (77 and 92%) and bone scan (86 and 88%) (45). 18F-FDG-PET/CT also showed better sensitivity for detecting bone metastases in patients with breast cancer than bone scintigraphy (96 vs. 76%) (46). Hybrid18F-FDG-PET/MRI, combining functional data of PET and superior soft tissue contrast resolution of MRI, has shown promising early results for detecting bone metastases and cancer staging and increased overall diagnostic confidence and lesion conspicuity (44), although there has not been widespread adoption of PET/MR scanners outside academic settings.

## Differential diagnosis

### Spinal epidural infection

**Figure 9:**
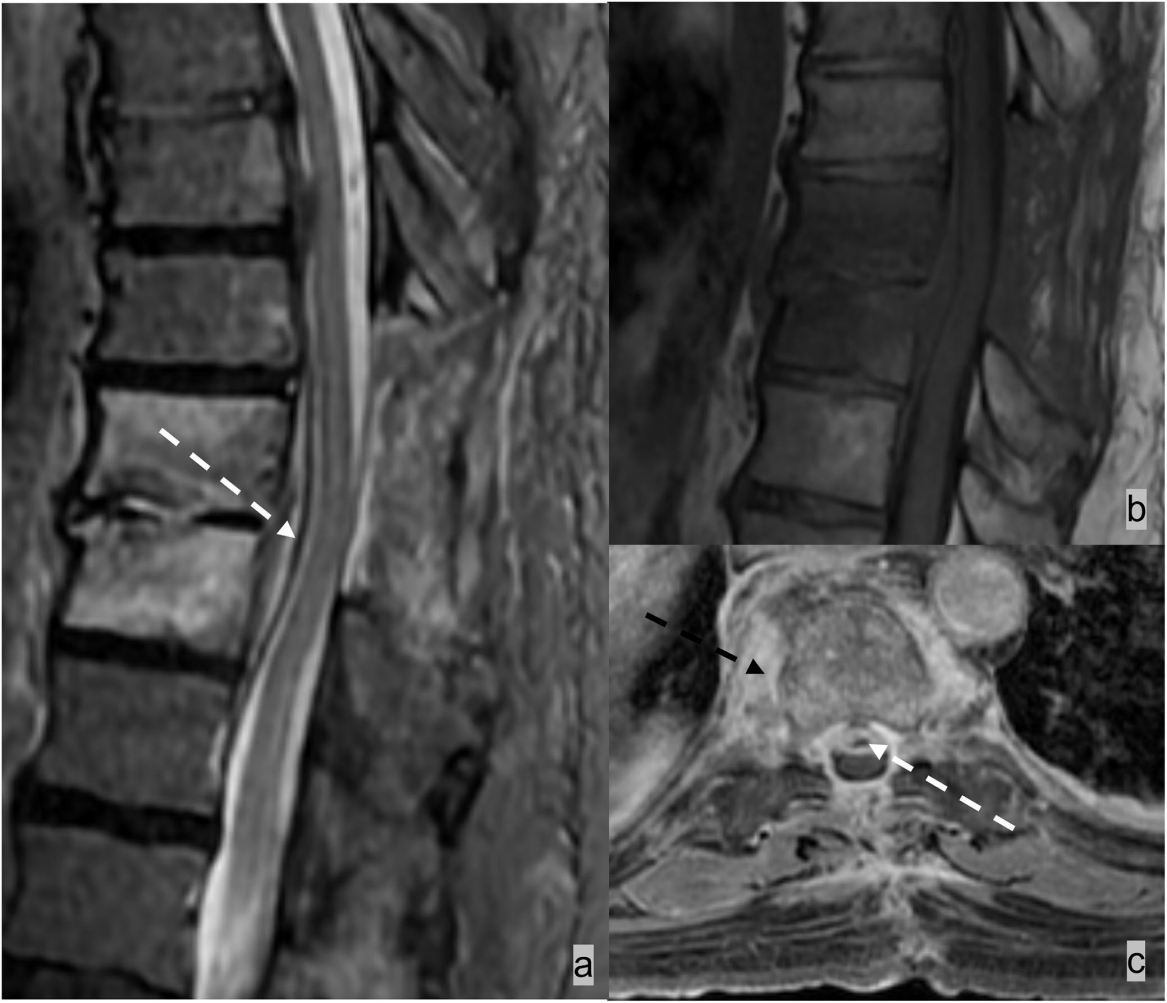
**(a,b)** Patient had prior T8-10 decompression laminectomy. STIR, sagittal T1W, and axial T1W post-contrast sequences demonstrated osteomyelitis/discitis at T9-10 with an enhancing ventral epidural abscess containing central nonenhanced necrosis (dotted white arrow). Paraspinal soft tissue involvement with phlegmon/inflammatory changes (dotted black arrow) is often seen in spinal infection **(c)**.

Spinal epidural infections typically occur as a direct extension of osteomyelitis and discitis, although primary epidural infections can occur after trauma, epidural injection, and surgery (47). The most common pathogen is *Staphylococcus aureus*. The thoracic and lumbar regions are the most common epidural abscess sites due to the relatively large epidural space (48). Epidural abscesses are more commonly located in the ventral epidural space in adult patients and in the dorsal epidural space in pediatric patients (49). Epidural infection is typically iso- to hypointense compared with the spinal cord on a T1W sequence and hyperintense on a T2W image (50). Epidural infections demonstrate three patterns of enhancement: diffuse enhancement of thickened and inflamed tissue and associated microabscesses and granulomatous material in an epidural phlegmon; peripheral enhancement with central nonenhanced debris representing epidural abscess; and a combination of both (Figure 9) (50). Typically, diffusion restriction indicates the presence of an epidural abscess. In addition, the presence of T2-hyperintensity in discs, vertebral bodies, and paravertebral soft tissues with possible paravertebral abscesses can help to distinguish epidural infection from epidural metastases. Additional clinical information is also important to make a diagnosis. Clinical presentation varies from asymptomatic to lumbago with sepsis (51). Predisposing risk factors include immunocompromised state, recent spine surgery or injection, and history of intravenous drug abuse (51). Laboratory findings include leukocytosis and elevated inflammatory markers such as erythrocyte sedimentation rate (ESR) (51).

### Spinal epidural hematoma

**Figure 10:**
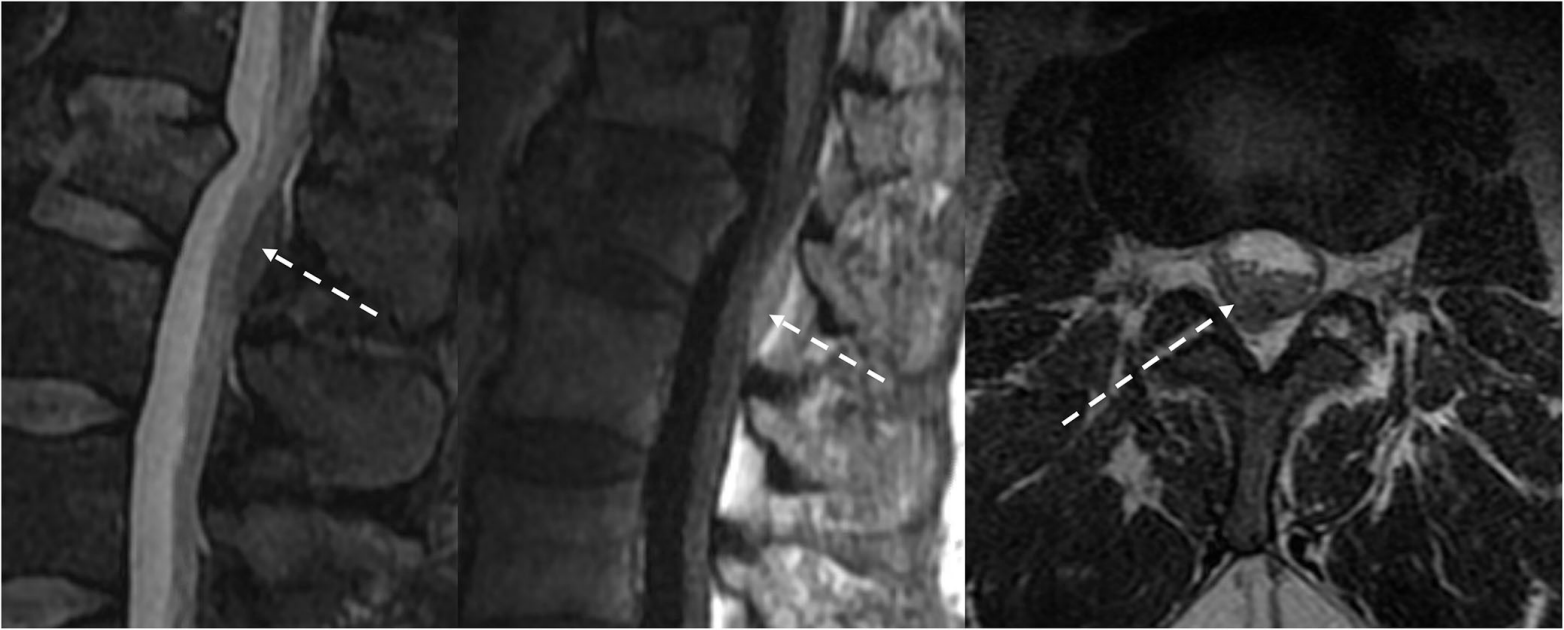
**(a,b)** MRI of the lumbar spine in this patient with traumatic injury demonstrated an L1 burst compression fracture and a small dorsal epidural hematoma at the L1-2 level. **(C)** The T1 hyperintense and T2 hypointense biconvex-shaped epidural hematoma effaces the epidural fat.

The epidural space is the most common site of spinal hematoma due to the abundant vascularity of the epidural space (52); 40% of epidural hematomas are idiopathic (53). Additional etiologies include trauma, coagulopathy, and iatrogenic injury from recent surgical or interventional procedures (52). Epidural hematomas most often develop in the dorsal epidural space (75%) than in the ventral epidural space because of tighter adherence of the dura to the posterior spinous ligament anteriorly than with ligamentum flavum posteriorly (52, 53). Epidural hematomas often have a biconvex-shaped collection with a well-delineated border effacing the epidural fat and inwardly displacing the thecal sac (Figure 10). The specific MRI signal characteristics of epidural hematoma vary according to the evolution of the oxidative state of hemoglobin over time but typically show a hypointensity on susceptibility-weighted sequences in acute, subacute, and chronic hematomas (52).

### Discogenic disease

**Figure 11:**
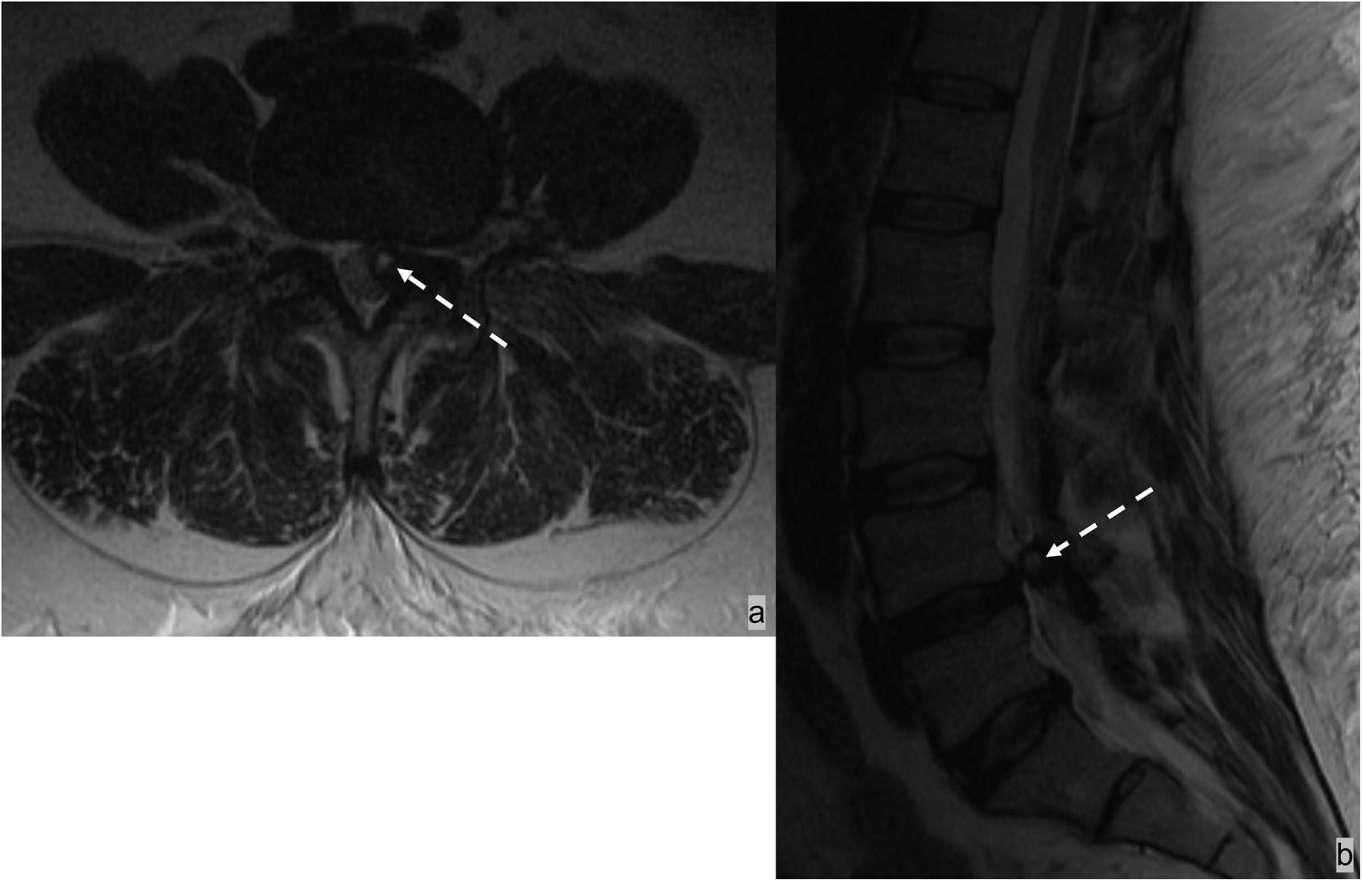
Patient presented with back pain radiating to the left lower extremity. MRI demonstrated a T2 hyperintense synovial cyst in the epidural space abutting the left facet joint at L4-5, which resulted in stenosis of the left subarticular recess and impingement of left L5 traversing nerve root.

Discogenic diseases, such as disc herniation, synovial cysts, and post-surgical epidural fibrosis, are epidural processes that may result in a mass effect on the thecal sac. These may mimic epidural metastases clinically and imaging appearance. Herniated disc contents, including disc protrusion, extrusion, and sequestration, have an epidural component with signal characteristics similar to the nucleus pulposus of the underlying disc. Continuity of the herniated disc material with the underlying disc can usually be identified with MRI, except for disc sequestration, which is discontinuous with the underlying disc. Synovial cysts are associated with facet arthropathy and are located adjacent to the facet joint. Although well-circumscribed, synovial cysts may demonstrate heterogeneous T1W and T2W signals because of internal debris and hemorrhage (Figure 11). Epidural fibrosis, which is often asymptomatic, is a consequence of post-surgical granulation tissue and scarring. Fibrosis is typically T1-isointense and T2-iso-hyperintense relative to disc material and often demonstrates immediate homogenous contrast enhancement (54).

### Other benign spinal epidural lesions

Spinal epidural hemangiomas are rare vascular malformations in the epidural space. Lee et al. described four types of epidural hemangiomas based on MR features and histology: type A is an arteriovenous type with a cystic-appearing mass with T1 hyperintense signal; type B is a venous type with a cystic-appearing lesion with T1 iso-intense signal; type C is a cavernous-type with a solid hypervascular lesion; type D is a cavernous-type with hematoma (55). Type C cavernous-type is most common. Regardless of the type, spinal epidural hemangiomas generally have a lobular contour and a T2 hypointense rim, which reflects a fibrous capsule or hemosiderin deposition (Figures 2a–c) (55).

Extramedullary hematopoiesis most commonly involves the liver, spleen, kidneys, and paraspinal soft tissue in the posterior mediastinum, and it is often associated with chronic anemia or myeloproliferative disorders (47, 56–58). Although rare, extramedullary hematopoiesis can involve the epidural space resulting in spinal cord compression (59). It has a well-defined lobulated contour and typically shows an iso-intense T1W signal to the spinal cord, variable T2 signal intensities, and absent or minimal enhancement (47). Spinal vertebrae may show diffusely abnormal loss of T1W in the affected marrow because of reconversion of normal fat-containing marrow into hematopoietic tissue (47).

## Epidural spinal cord compression scale

The Neurologic, Oncologic, Mechanical, and Systemic (NOMS) framework is a multidisciplinary guideline developed by Memorial Sloan-Kettering Cancer Center to characterize the clinical, imaging, and histologic features of malignant lesions to guide the therapeutic approach for patients with spinal metastatic tumors (60). The neurologic assessment of the NOMS paradigm focuses on the degree of spinal cord compression, and includes radiographic assessment and clinical evaluation of myelopathy and radiculopathy. The Epidural Spinal Cord Compression (ESCC) Scale serves as a guideline for radiographic assessment (Table 1). It is a 6-point scale using axial T2W sequence on the most severe cord compression site to determine the degree of epidural metastatic spinal cord compression and guide management (19, 37, 60). Grades 0, 1a, and 1b are usually considered appropriate for radiation therapy as initial treatment in the absence of mechanical instability. Grades 2 and 3 are considered high-grade ESCC and require surgical decompression before radiation unless the tumor is known to be highly radiosensitive (37, 60). Management of Grade 1c is controversial and may involve high-dose hypofractionated radiation or stereotactic spine radiosurgery (61).

**Table 1:** Epidural spinal cord compression scale (ESCC) (35).

Grade 0: Osseous disease only Grade 1a: Epidural involvement without thecal sac deformation Grade 1b: Thecal sac deformation without cord contact Grade 1c: Thecal sac deformation with cord contact Grade 2: Cord compression with preservation of some CSF Grade 3: Cord compression with complete effacement of CSF

## Spinal instability neoplastic score

The Spinal Instability Neoplastic Score (SINS) is a guideline to identify spinal instability in patients with spinal metastases (Table 2) (62, 63) and is used in conjunction with the ESCC Scale to guide management. SINS consists of 5 imaging parameters: location, bone lesion type, spinal alignment, vertebral body collapse, and posterolateral spinal element involvement. Mechanical pain is the only clinical parameter considered (62, 63). A cumulative score ranging from 0 to 18 reflects the severity of spinal instability, which stratifies patients into stable (0–6 points), potentially unstable (7–12 points), and unstable (13–18 points). In a patient with a score of 7 and above, surgical consultation is warranted (62).

**Table 2:** Spinal instability neoplastic score (SINS) (58).

Location	
Junctional spine (occiput- C2, C7-T2, T11-L1, L5-S1)	3
Mobile spine (C3-6, L2-4)	2
Semirigid spine (T3-10)	1
Rigid spine (S2-5)	0
Alignment	
Subluxation or translation	4
New kyphosis or scoliosis	2
Normal alignment	0
Vertebral body involvement	
>50% collapse	3
<50% collapse	2
No collapse, but >50% involved	1
None	0
Posterior element involvement	
Bilateral	3
Unilateral	1
None	0
Lesion quality	
Lytic	2
Mixed	1
Blastic	0
Pain	
Yes, mechanical	3
Occasional nonmechanical	1
None	0

## Conclusion

Metastatic epidural spinal cord compression is a common and potentially devastating manifestation of systemic metastatic disease and is becoming an increasingly common oncological emergency as the average age of populations continues to increase in many developed countries. Imaging is central in the diagnosis and evaluation of the extent of spinal metastatic disease, and can accurately determine the involvement of the epidural space and the degree of spinal cord compression if present. A compartmental approach to assessing and describing imaging features helps to refine the differential diagnosis and contributes to optimal management. Familiarity with established grading scales, including NOMS, ESCC, and SINS, is important to appropriately categorize clinically relevant imaging features of metastatic disease of the spine and to allow for a meaningful contribution to appropriate management.

## Author contributions

JB, DK-O'C, AH, and KG contributed to the case collection for this review paper. JB wrote the manuscript. All authors contributed to manuscript revision, read, and approved the submitted version.

## Conflict of interest

The authors declare that the research was conducted in the absence of any commercial or financial relationships that could be construed as a potential conflict of interest.

## Publisher's note

All claims expressed in this article are solely those of the authors and do not necessarily represent those of their affiliated organizations, or those of the publisher, the editors and the reviewers. Any product that may be evaluated in this article, or claim that may be made by its manufacturer, is not guaranteed or endorsed by the publisher.
